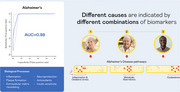# Early detection and management of Alzheimer's Disease through multi‐omic analysis of blood

**DOI:** 10.1002/alz70856_105281

**Published:** 2026-01-08

**Authors:** Mohammad Anwar, Brandon Landry, Windy Wang, Karm Alhasan, Haiyan Yang, Robert Fraser

**Affiliations:** ^1^ Molecular You, Vancouver, BC, Canada

## Abstract

**Background:**

Alzheimer's disease (AD), a leading cause of dementia, poses a global health challenge as aging populations expand. Traditionally, practitioners rely on symptom‐based identification, often miss the critical window for early intervention, limiting opportunities for improved clinical outcomes. Molecular You (MY) has developed an innovative, two‐sided platform to address this gap. The platform i) quantifies 280 high‐value plasma metabolites and proteins with absolute quantification and ii) through its algorithms ranks health risks and provides insights across more than 20 biological systems and pathways with an average predictive value of 88%.

This study demonstrates the value of applying the MY platform in identifying AD risk and associated co‐morbidities early, stratifying patients into endotype‐specific mechanisms that inform customized care plans.

**Method:**

Plasma samples from 74 patients, including 5 diagnosed with AD, were collected after an overnight fast, rapidly frozen, and shipped to MY for analysis. Each specimen underwent quantitative metabolomic (143 metabolites) and proteomic (140 proteins) assays using well‐established LC‐MS/MS methods. The resulting data were processed through the MY platform to identify health risks and map key biological pathways.

**Result:**

Four of the five patients diagnosed with AD were confirmed, while one patient's profile was more aligned with vascular dementia. Additionally, 36 patients were identified at moderate to high risk for AD. Analysis of the biological pathways revealed several distinct endotypes for AD risk: dyslipidemia/ abnormal lipid metabolism, metabolic aberrations, inflammation and oxidative stress driving neuroinflammation and neurodegeneration, and neurotransmitter dysfunction leading to excitotoxicity and synaptic dysfunction. Several co‐morbidities were identified in the population, including metabolic health issues such as diabetes, kidney health, liver health, immune health, cardiovascular disease, and inflammatory bowel disease. Personalized care plans were developed based on these findings, incorporating targeted dietary, lifestyle, and clinical interventions.

**Conclusion:**

The MY platform demonstrates the feasibility of multi‐omic analysis of blood to enhance the early detection of AD before clinical symptoms manifest, identify emerging co‐morbidities, stratify patients into AD endotypes enabling precision preventative and personalized care. By monitoring patients longitudinally using the MY platform it will be possible to objectively quantify treatment efficacy, safety and overall patient outcomes.